# Supplementary material for: Are carbon emissions trading and green financial instruments synergistic? -Comprehensive quantitative research based on content analysis
Source: PLoS One. 2024 Mar 7;19(3):e0298601. doi: 10.1371/journal.pone.0298601 (PMC10919601; doi:10.1371/journal.pone.0298601)
Supplement: S1 Data — (ZIP) [file pone.0298601.s001.zip › Data/地级市Z_F工作报告.zip!/╣π╢1⁄2/╔ε█┌╩╨/└┤╫╘╣1⁄2╓┌║┼í╛┬φ┐╦╩2╛▌═°í┐_2017.docx]

各位代表：

　　现在，我代表深圳市人民政府向大会作政府工作报告，请予审议，并请各位政协委员和其他列席人员提出意见。

　　一、2016年工作回顾

　　过去一年，是全市上下紧密团结在以习近平同志为核心的党中央周围，践行新发展理念，增创“四个全面”新优势，实现“十三五”良好开局的一年。我们在党中央国务院、省委省政府和市委的坚强领导下，全面贯彻党的十八大和十八届三中、四中、五中、六中全会精神，深入落实习近平总书记系列重要讲话和对深圳工作的重要指示批示精神，坚持稳中求进工作总基调，着力推进供给侧结构性改革，持续打造深圳质量、深圳标准，扎实开展“城市管理治理年”工作，加快建设现代化国际化创新型城市，实现了有质量的稳定增长、可持续的全面发展，完成了市六届人大二次会议确定的目标任务。

　　（一）质量型发展领先优势更加凸显。始终坚持质量第一、以质取胜，以质量优势对冲了成本劣势，以质量先行赢得了发展主动。主要经济指标“稳中走强”。有效应对外部经济下行压力，实现GDP增速逐季攀升、逆势上扬。初步核算，2016年本市生产总值19492.6亿元，同比增长9%；固定资产投资4078.2亿元，增速达23.6%、创22年新高；社会消费品零售总额5512.8亿元，增长8.1%。质量效益“好中更优”。在全面落实各项税费减免政策的基础上，辖区公共财政收入7901亿元，其中地方一般公共预算收入3136亿元，增长15%，四年翻了一番多。规模以上工业企业利润增长10%。资源能源消耗“低中再降”。预计万元GDP能耗、水耗分别再下降4.1%和8.9%，化学需氧量、氨氮、二氧化硫、氮氧化物排放量分别下降8.6%、5.2%、10.2%和3.4%，PM2.5平均浓度为27微克/立方米，下降10%，继续处于国内城市领先水平，实现了经济质量和生态质量“双提升”。

　　（二）供给侧结构性改革精准发力。继续从质量引领、创新驱动、转型升级等方面发力供给侧，制定实施供给侧结构性改革总体方案和5个专项行动计划，进一步在提高供给质量上下功夫，在增强供给能力上出实招，不断增创供给侧新优势。精准化组合型政策激发供给活力。突出供给侧关键环节，出台科技创新、企业竞争力、人才优先发展、人才住房、高等教育等“一揽子”政策，集中推出235条措施。设立重大产业、军民融合、并购、中小微企业发展等系列基金，总规模超过2000亿元，引导更多资金向有效供给配置。全年工业投资、工业技改投资分别增长17.1%、15.3%，民间投资增长61.5%。新增商事主体56万户，增长26.2%，累计达265万户。降成本补短板释放供给潜力。开展降低实体经济成本行动，实施“营改增”、调整“五险一金”、取消15项涉企行政事业性收费等政策措施，政府回购龙大、南光、盐排、盐坝4条高速公路并免费通行，全年为企业减负超过1200亿元，比上年多减负500亿元。全面加大补短板力度，在教育、医疗、交通、公共安全等重点领域实施159个项目，完成投资927亿元。高标准高质量提升供给水平。继续推进标准、质量、品牌、信誉一体化建设，实施代表高品质的“深圳标准”认证标识，创建“标准国际化创新型城市”，主导或参与制定国际标准249项，增长53.7%。华为、中兴分别荣获中国质量奖和中国工业大奖，腾讯等3家企业入选2016“全球最具价值品牌百强榜”。

　　（三）发展新动能强劲迸发。率先构建综合创新生态体系的效应不断显现，创新加快从跟跑向并跑领跑迈进，深圳建设科技、产业创新中心的定位纳入国家“十三五”规划。创新能力加快提升。全社会研发投入超过800亿元，占GDP比重提高至4.1%。国家基因库投入运营，成为全球最大的基因库之一。新增国家省市级重点实验室、工程实验室、工程研究中心、企业技术中心等创新载体210家，累计达1493家。新组建神经科学研究院等新型研发机构23家。PCT国际专利申请量增长约50%，占全国一半。国内发明专利申请量增长约40%。荣获国家科技奖励16项和中国专利金奖、外观设计金奖4项，其中深圳企业参与的4G TD-LTE关键技术与应用首获国家科技进步奖特等奖。华为短码方案成为全球5G技术标准之一。石墨烯太赫兹芯片、无人机、柔性显示等技术处于全球领先水平。开放创新布局提速。设立两家由诺贝尔物理学奖、化学奖获得者领衔的科学家实验室，建立深圳—密歇根贸易、投资和创新合作中心，苹果、微软、高通等全球知名科技企业在深设立研发机构。总规模2000亿元的中国国有资本风险投资基金落户深圳，VC/PE机构累计近5万家、注册资本约3万亿元。“孔雀计划”产业园和市人才研修院，举办首届海外创新人才大赛，新引进全职院士6名、高层次创新团队23个，新增海归人才1.05万人、高技能人才12.6万人。创新型经济快速发展。战略性新兴产业和未来产业增加值增长10.6%，其中互联网产业增长15.3%，机器人、可穿戴设备和智能装备产业增长20.2%。无人机、新能源汽车等新产品产量分别增长60%和146%。新增国家级高新技术企业2513家，是上年增量的3倍以上，累计达8037家。创新创业活力全面激发。全国“双创”周主会场活动及第二届深圳国际创客周成功举办，吸引35个国家和地区50万人次参加。新增创客空间81家、创客服务平台32家、孵化器12家，7000余台大中型科研仪器设备向社会开放共享，深圳湾创业广场成为全国创新创业的新名片。高交会、国际人才交流大会、IT和BT领袖峰会等平台功能不断提升，南山区、招商局集团入选首批国家级“双创”示范基地，深圳成为国家小微企业创业创新基地示范城市。

　　（四）优质高端产业加速壮大。充分发挥转型升级的先行优势，瞄准更高目标，继续优化产业结构，构建更加强大的现代产业体系。全年支持新兴产业项目1032个，支持服装、家具、钟表、黄金珠宝、眼镜等优势传统产业升级项目456个，淘汰转型低端落后企业1400多家。产业结构凸显“三个为主”。经济增量以新兴产业为主，新兴产业对GDP增长贡献率提高至53%左右；工业以先进制造业为主，先进制造业占工业比重超过75%；三产以现代服务业为主，服务业占GDP比重60.5%，现代服务业占服务业比重提高至70%以上，其中金融业增加值增长14.6%，新增玉山银行等持牌法人金融机构13家。高端产业项目加快落地。国际生物谷坝光核心区、柔宇国际柔性显示基地、光启未来科技城等园区启动建设。华星光电第11代液晶面板、中芯国际12英寸芯片、开沃新能源汽车、国际会展中心等150个重大项目开工，总投资超过1000亿元。总部经济效应不断增强。本土世界500强企业增至5家，营业收入超五百亿元企业达22家，其中超千亿元企业11家。境内外上市公司增加32家，累计达346家。新兴际华国际总部、发那科南方总部等落户深圳，平安国际金融中心、深圳阿里中心、百度国际总部等建成启用。

　　（五）绿色发展质量进一步提升。坚持久久为功，建设绿色家园，大力实施大气质量提升40条、治水提质40条等系列政策措施，宜居宜业的环境优势不断彰显。“深圳蓝”成为靓丽名片。空气优良天数提高至354天，居内地城市最优水平。全面禁行黄标车，淘汰老旧车及黄标车近7万辆。新推广应用新能源汽车2.9万辆，总量超过6.7万辆，新建充电桩7000个以上。靠港船舶全部强制使用低硫燃油，累计建成覆盖12个泊位的岸电设施。绿色建筑面积增长61%，总量达5320万平方米。碳交易扩大至交通领域，管控企业增加至824家。开工建设东部环保电厂、老虎坑三期、妈湾能源生态园等环保基础设施，全面推进现有垃圾焚烧厂、填埋场提升改造。创建垃圾分类和减量达标小区400个。绿色生态空间持续增加。新建、改造公园61个，罗湖体育休闲公园等投入使用，国际友好城市公园、坝光银叶树湿地公园等加快建设。建成绿道“公共目的地”380个，规划建设300公里“远足径”，推进深圳湾滨海休闲带西段等建设，打造海滨最美风景线。治水提质效果初显。坚持以流域为单元实施系统治理，全年投入111亿元，启动461个治水项目，15条主要河流中有10条水质改善，建成区黑臭水体减少35.6%，新增污水管网1033公里，消除479个排污口，整治83公里河道和142个内涝点。开展茅洲河综合整治“百日大会战”，开工沙井水质净化厂二期等一批项目。深圳湾污水截排二期工程、福田水质净化厂建成使用。环保执法力度不断加大。认真办理中央环保督察组交办案件，办结259件、立案处罚162件，切实解决一批环保突出问题，做到事事有回应、件件有落实。全年查处环保违法行为1688宗，停产整治企业61家。

　　（六）城市管理治理成效显著。坚持安全发展，开展安全生产16项专项整治，落实基础设施建设五年计划，实施拓展空间十大行动，着力治理城市顽疾、完善城市功能。安全生产和公共安全全面加强。深刻吸取光明新区“12?20”滑坡事故教训，逐项抓好国务院事故调查报告意见整改落实，强化安全生产责任，完善公共安全白皮书，着力以最严格的标准、制度、处罚和问责，保障市民生命财产安全和城市安全运行。单独设立市安监局，组建城市公共安全技术研究院，设立150亿元专项资金，建立3320人的监督检查员队伍，健全57个专项应急预案。全面推进余泥渣土受纳场、危险边坡和地质灾害等重点隐患排查整治，完成隐患整改69.1万项，关闭取缔企业、场所3968个。清水河、东角头油气库加快迁移，二线“插花地”棚户区改造正式启动。成功应对“妮妲”“海马”等强台风袭击。完善社会治安防控体系，110接报刑事治安警情下降25.3%，刑事案件破案宗数提高10.7%。严厉打击食品药品违法行为，食用农产品抽检批次增加80%，国家食品安全城市创建取得新进展。违法建筑实现“零增量”。落实严查严控违建“1＋2”文件，实行严格的区党政一把手考核问责制，坚持以拆控停、疏堵结合，完善查违执法机制，开展全天候巡查防控，累计拆除消化违建约1100万平方米，实现违建增量“零增长”、存量“负增长”。现代化基础设施加快完善。对外战略通道进一步打通，新开通国际航线12条，机场旅客吞吐量突破4000万人次，太子湾国际邮轮母港开港运营，赣深客专开工，深中通道、外环高速等加快建设。新一轮轨道交通建设全面推开，开工建设6、8、10号线及2、3、4、5、9号线等8条延长线，7、9、11号线开通，地铁运营里程达285公里。道路交通网络不断优化，南坪快速三期、坪盐通道、坂银通道等建设有序推进，春风隧道、沙河西路快速化改造等启动，41条断头路打通，15个原二线关口交通改善项目完工。427条定制化巴士线路和46条社区微巴线路开通。信息基础设施更加完善，完成城域网IPv6升级改造，新建移动通信基站1.45万座，光纤入户率由43%提高至80%。区域发展布局优化。东进战略启动实施，出台五年行动方案，计划安排355个重点项目、总投资1.65万亿元，横坪公路改造等51个先行项目开工建设。行政区划调整取得突破，龙华区和坪山区获国务院批复设立，宝安、龙岗等4个区街道拆分，为城市精细化管理和特区一体化提供有力支撑。深圳湾超级总部基地等16个重点区域完成年度投资940亿元。原特区外投资超过2500亿元。资源保障能力提升。推进城市更新和土地整备，全年更新供应土地2.2平方公里，整备释放土地11平方公里。科学用海力度进一步加大。公明供水调蓄主体工程完工，清林径引水调蓄工程加快推进，坂雪岗、光明水厂投入使用。建成136个优质饮用水入户工程、惠及居民15万人，完成124个原特区外社区供水管网改造。

　　（七）改革开放全面推进。坚持改革不停顿、开放不止步，继续推进十八届三中全会以来主动承接的中央和省242项改革试点，重点抓好39项年度改革任务，努力种好改革开放“试验田”。强区放权迈出新步伐。下放城市更新、产业用地出让等重点领域事权，调整规划国土、交通等驻区部门为市区双重管理，下放充实基层编制2374名。全面推广罗湖城市更新改革试点经验，审批时间缩短2/3。重点领域改革实现新突破。制定深化医药卫生体制改革实施意见和社会办医政策措施，优化调整公立医院医疗服务价格。推广罗湖医改试点，组建基层医疗集团。新一轮国资国企改革启动，教育、户籍制度、地税征管方式、国有文化集团、警务辅助人员管理等改革加快推进，“深港通”正式开通。前海蛇口自贸片区建设呈现新面貌。新推出制度创新举措106项，其中36项全国领先，23项全省复制推广。新增企业5.3万家，累计达12.5万家，注册企业增加值增长38%。前海深港合作提速，港交所前海联合交易中心、汇丰集团华南总部、首家港资控股基金公司等1894家港企落户，累计77个香港创业团队入驻深港青年梦工场。自贸新城建设加快，平南铁路拆迁、航空限高等瓶颈问题取得突破，全年完成固定资产投资370亿元，增长37.7%。对外开放再上新台阶。积极落实“一带一路”倡议，粤港澳大湾区纳入国家“十三五”规划，中白物流园等境外园区建设加快，“一带一路”环境技术交流与转移中心落户深圳，巴布亚新几内亚深圳产品展销中心成立运营，对沿线国家协议投资额增长11.5%。着力优化外贸结构，新出台出口退税周转金贴息等19条措施，全年进出口总额2.63万亿元，其中出口继续居内地城市首位，一般贸易占比提高3.2个百分点，外贸综合服务企业、跨境电商进出口额分别增长15%和17%。合同利用外资、实际对外投资分别增长103%和70%。新增多伦多、麦纳麦等10个国际友城。与港澳在科技教育、文化创意、检验检疫等领域合作进一步加强，落马洲河套地区深港合作开发启动，莲塘/香园围口岸联检楼封顶。对口支援和区域合作取得新进展。坚持精准扶贫、精准脱贫，扎实推进援疆援藏和对口帮扶河源汕尾、贵州毕节、四川甘孜、重庆巫山等，启动对口广西百色、河池扶贫协作，完成湖南湖北三县贫困人口劳务输出对接试点任务，全年安排财政帮扶资金62.5亿元，增长31.6%。深汕特别合作区加快建设，深河、深汕共建产业园区累计投产项目321个，中兴通讯河源项目、比亚迪汕尾生产基地等顺利推进，深圳至惠州、汕尾捷运线开通，深莞惠“3＋2”经济圈建设提速。

　　（八）补短板惠民生力度不断加大。坚持发展为民、发展惠民，进一步加强和改善公共服务供给，九大类民生支出2380.3亿元，其中教育、医疗支出分别增长32.3%、33.7%。116件民生实事和7716件民生“微实事”全面完成，12项重大民生工程年度投资超过300亿元。社会保障水平稳步提升。新增就业10.3万人，城镇登记失业率2.3%，社会保险参保人次增长6%。建立困境儿童基本生活保障制度。建成市社会福利中心一期工程和市养老护理院，新增老年人日间照料中心25家。教育事业发展取得突破。高考一本、本科上线率均居全省首位，新增普惠性幼儿园98所、幼儿园学位2.5万个、公办中小学学位3.1万个。高等教育开放发展全面提速，与北京大学、清华大学等6所国内名校签约建设深圳校区，深圳北理莫斯科大学获批成立，中山大学深圳校区、哈尔滨工业大学（深圳）招收首批本科生，天津大学佐治亚理工深圳学院等10所特色学院建设加快。深圳技术大学、中德先进制造技师学院启动筹建。医疗卫生事业快速发展。大力实施“三名工程”，引进高水平医学团队50个，新建名医诊疗中心5家，建成萨米国际医疗中心（深圳）。中国医学科学院肿瘤医院深圳医院获批。增加三级医院6家、三甲医院1家，新增病床3000张、执业医师2698名。完成社康中心基本设备标准化配置，家庭医生签约服务人数新增62.9万名。住房保障不断加强。坚决贯彻国家房地产市场调控政策，先后出台“深六条”和“深八条”，促进房地产市场平稳健康发展。成立注册资本1000亿元的市人才安居集团，新开工及筹集人才和保障性住房6.2万套、竣工5.1万套、供应4.2万套，均为上年的2倍以上。新引进人才一次性租房和生活补贴标准提高至本科1.5万元、硕士2.5万元、博士3万元。文体等事业繁荣发展。首届国际博物馆高级别论坛在深召开，当代艺术与城市规划展览馆竣工，第十二届文博会实际成交额增长23.4%。全年获德国iF设计奖、红点奖共140项，比上年增长50.5%。举办ATP和WTA国际网球公开赛、深圳国际马拉松赛、中国杯帆船赛等高水平赛事，与拜仁慕尼黑俱乐部签约共建国际足球学校，335所公办中小学体育场馆向社会开放，深圳运动员在里约奥运会上取得“一金两银”的优异成绩。新增专业社工1300人。连续第六次获“全国双拥模范城”称号。此外，信访、统计、计生、民族、宗教、对台、侨务、民防、档案和国防动员等工作取得新成绩。

　　（九）政府职能作风双转变深入推进。认真贯彻十八届六中全会精神，把全面从严治党要求切实体现到政府工作中，扎实开展“两学一做”学习教育，着力转职能、转作风、提效能，努力建设为民务实清廉政府。严格依法履职。坚决执行法律法规和市人大及其常委会决议决定，自觉在市人大、市政协监督下开展工作，提请市人大审议法规草案4件，立改废政府规章10件，办理人大代表建议818件、政协提案572件。编制“十三五”规划纲要及51个专项规划。出台市政府重大行政决策程序规定和部门权责清单管理办法，制定水污染治理等3个专项权责清单。规范委托执法行为，加强食品安全等重点领域专项执法。全面启动“七五”普法，提升全社会法治意识。加大“放管服”力度。取消转移下放293项市级行政职权，清理规范53项行政职权中介服务事项。推行“双随机、一公开”，加强事中事后监管。创新政府服务模式，在企业设立、人才服务、民政等领域推行“一窗式”服务，将网上办事大厅延伸至街道、社区，行政审批事项网上全流程办理率和办结率均超过97%。加强廉政勤政建设。认真执行廉洁自律准则、纪律处分条例等规定，保持惩治腐败高压态势，查处了一批违法违纪案件。严格落实中央八项规定精神，深入整治“四风”，严控“三公经费”。加大审计监督力度，全面整改审计发现的问题，市本级部门预算执行审计实现全覆盖。加强公务员队伍建设，出台支持改革创新建立容错纠错机制的若干规定，营造鼓励干事创业的良好环境。

　　各位代表，过去一年，我们牢固树立“四个意识”，以习近平总书记系列重要讲话和对深圳工作的重要指示批示精神为根本遵循，攻坚克难、乘势而上，在“十三五”新起点上实现了新发展，取得了来之不易的成绩。这是在党中央国务院、省委省政府和市委正确领导下，在市人大及其常委会和市政协监督支持下，全市人民凝心聚力、共同奋斗的结果。在此，我代表深圳市人民政府，向全市人民，向给予政府工作大力支持的人大代表和政协委员，向各民主党派、工商联、各人民团体和社会各界人士，向中央和省各部门、中央和各省区市驻深单位，向驻深解放军、武警官兵，向关心和支持深圳改革开放创新的港澳同胞、台湾同胞、海外侨胞和国际友人，表示崇高的敬意和衷心的感谢！

　　同时，我们也清醒地认识到，深圳经济社会发展还存在不少问题、面临诸多挑战。一是生产要素成本上升过快，对产业加快迈向中高端提出更加紧迫的要求。二是基础研究机构较少，创新领军人才、高技能人才不足，难以满足国际科技、产业创新中心建设需要。三是特区快速发展中积累的违法建筑、水体污染等问题依然突出，垃圾处理能力严重不足，公共安全基础还不牢固。四是城市建设管理标准和精细化程度不高，与国际一流城市相比还有差距。五是公共服务供给还存在不少短板，优质教育、医疗等资源缺乏，部分区域和时段交通拥堵。六是政府职能转变还不到位，行政效率仍有较大提升空间，一些公职人员干事创业劲头不足、责任担当意识不强，违法违纪问题时有发生。我们要直面问题，把市民的期盼作为努力的方向，把问题的解决作为检验工作成效的标准，勇于担当、迎难而上，推动特区各项事业迈上新台阶。

　　二、2017年主要工作

　　2017年是率先全面建成小康社会的攻坚年，是供给侧结构性改革的深化年，将迎来党的十九大胜利召开。作为经济特区，我们要充分发挥中国特色社会主义制度的巨大优越性，抢抓改革时代、创新时代、质量时代的新机遇，坚持变中求新、新中求进、进中突破，勇当改革的弄潮儿、创新的引领者、质量的新标杆，努力以特区的使命担当创造“四个全面”新业绩，打造彰显“四个自信”新样板。

　　今年政府工作的总体思路是：紧密团结在以习近平同志为核心的党中央周围，认真贯彻党的十八大以来中央各项决策部署，全面落实习近平总书记系列重要讲话和对深圳工作的重要指示批示精神，按照省委省政府和市委的部署要求，紧紧围绕“五位一体”总体布局和“四个全面”战略布局，坚持稳中求进工作总基调，坚持以新发展理念引领新常态，坚持以推进供给侧结构性改革为主线，坚持深圳质量、深圳标准，加快建设现代化国际化创新型城市和国际科技、产业创新中心，促进有质量的稳定增长、可持续的全面发展，以优异成绩迎接党的十九大和省第十二次党代会胜利召开。

　　供给侧结构性改革是中国特色社会主义政治经济学的重大创新，也是深圳新时期构建长远竞争力的根本战略。做好今年的工作，我们要突出重点、抓住关键，扎实开展“城市质量提升年”工作，紧紧扭住供给侧结构性改革这一主线，努力争当供给侧结构性改革排头兵。

　　争当供给侧结构性改革排头兵，就是要继续强化深圳质量领先优势，率先打造高质量的供给体系。在成功实现“深圳速度”向“深圳质量”跨越的基础上，始终坚持质量主攻方向，树立质量第一的强烈意识，进一步将大质量大标准渗透到经济社会发展各领域和全过程，不断深化标准、质量、品牌、信誉一体化建设，着力以标准提升供给品质，以质量创造供给价值，以品牌塑造供给形象，以信誉拓展供给市场，推动供给体系质量不断升级，努力在国际经济大循环、世界供给大市场中赢得主动。

　　争当供给侧结构性改革排头兵，就是要继续强化创新驱动的先发优势，率先形成国际一流的供给能力。充分利用较早实现动力转换的有利条件，加快建设国际科技、产业创新中心，前瞻布局颠覆性技术、先导性产业，勇当引领式创新先锋和新经济领跑者，以创新优化供给方式、提升供给效率，以创新突破新技术、创造新供给、催生新需求，推动供给能力全面跃升，不断巩固提升以创新为鲜明特征的供给优势。

　　争当供给侧结构性改革排头兵，就是要继续抢抓转型升级的先机，率先构建满足需求升级的供给结构。围绕需求链部署产业链，把握消费从模仿型排浪式向个性化多样化转变的趋势，大力发展定制式制造、网络化生产，既以新兴产业发展扩大增量供给，又以传统产业升级优化存量供给，使产业结构跟上需求变化，使供给侧跑赢消费端，不断增强供给结构的适应性和灵活性，实现更高水平的供需动态平衡。

　　争当供给侧结构性改革排头兵，就是要继续发力实体经济“主战场”，率先厚植更加坚实的供给侧根基。大力弘扬企业家精神和工匠精神，把发展高端制造、精密制造、智能制造作为突出任务，把增强企业竞争力和活力作为关键路径，把扩大高质量投资和降低营商成本作为重要抓手，引导虚拟经济更好服务实体经济发展，推动更多资源要素向实体经济配置，打造更加强大的实体经济。

　　争当供给侧结构性改革排头兵，就是要继续勇担改革开放的特区使命，率先营造更加市场化法治化国际化的供给环境。始终坚持社会主义市场经济改革方向，狠抓改革攻坚和高水平开放合作，着力以改革破除供给约束、降低交易成本、优化制度供给，着力以开放集聚高端资源、提升配置能力、拓展供给空间，形成有利于供给要素高效配置的体制机制，全面激发供给活力。

　　争当供给侧结构性改革排头兵，就是要继续加大补短板惠民生力度，率先扩大高水平的公共服务供给。践行以人民为中心的发展思想，牢牢把握满足人民需求这一最终目的，坚持发展目的和发展过程的辩证统一，突出问题导向、需求导向，既立足当前加快补齐教育、医疗、水环境等民生短板，又面向未来有序扩大更高质量的公共产品和服务，更好地满足人民群众日益增长、不断升级的物质文化需要，努力在共建共享中增强市民获得感，在协调均衡中开拓发展新空间。

　　今年经济社会发展的主要预期目标是：本市生产总值增长8.5%左右，居民人均收入与经济增长基本同步，城镇居民登记失业率控制在3%以内，居民消费价格涨幅3%左右。需要说明的是，8.5%左右的增速是稳中求进的目标，是需要付出艰苦努力才能实现的目标，既考虑了深圳质量型经济发展的良好态势和创新驱动、转型升级的先发优势，又充分考虑了外部环境不确定性增多、各种潜在风险交织等不利因素。实现这一目标，既要把握“稳”的大局和主基调，把风险研判得更深入，把困难估计得更充分，把应对措施准备得更周全；更要在“进”上下功夫，积极进取、奋发有为，力争取得更好结果，为全国全省发展作出更大贡献。今年重点做好十个方面工作：

　　（一）统筹推进供给侧结构性改革和重点领域改革，释放经济社会发展新潜能。要在全面深化改革中突出供给侧结构性改革主线，坚持以改革破难题、促发展，进一步提高改革的精准性、系统性、有效性，增创体制机制新优势。一是着力加大实体经济支持力度。抓好科技创新、企业、人才等五大政策落地，放大政策叠加效应。支持民营经济发展，实施“专精新特”中小企业培育工程。制定完善土地供应的若干意见，加强对工业、创新型企业等用地保障。提高金融服务实体经济能力，积极建设风投创投中心，出台鼓励VC/PE投向初创期、种子期公司的若干措施，加大面向中小微企业的担保增信、风险补偿、贷款贴息等政策支持，探索成立科技保险机构。持续推进补短板、降成本，实施补短板项目245个、年度投资950亿元，全年为企业减负超过1350亿元。二是着力扩大高质量产品和服务供给。深入推进打造“深圳标准”行动计划，支持行业协会等制定更高的团体标准、行业标准。大力实施品牌竞争力、消费品工业质量、出口商品品质、工业设计能力四大提升工程，建设3个国家级质量检验检测中心，创建时装、钟表、机器人等知名品牌示范区。三是着力推进基础设施供给侧结构性改革。出台专门实施方案，探索组建基础设施金融机构，建立健全PPP政策体系，吸引更多社会资本参与交通、环保、综合管廊等设施建设，加强科技、质量、信息等新型基础设施供给，全年基础设施投资超过1100亿元，其中社会投资占比超过五成。四是着力以简政放权提高供给效率。继续大力推进强区放权，统筹第五轮市区财政体制和政府投资事权、市区职能调整等改革，增强市级决策统筹和区级执行落实能力，实现重心下移、财力下沉、权责匹配、效能提升。深化行政审批制度改革，再精简一批行政职权事项，推进投资项目“多审合一、多评合一”。五是着力以国资国企、商事制度等改革激发供给主体活力。实施市属国企改革“1＋12”政策措施，深化混合所有制改革，推动资源整合和并购重组，做强做优做大一批国企集团。推进社区股份公司转型发展。深化商事制度改革，推广“多证合一、一照一码”登记模式，强化事中事后监管，全面实施“双随机、一公开”。加快社会信用体系建设，加强信用信息采集、共享、使用，建立联动响应、联合惩戒机制，降低制度性交易成本。六是着力以社会事业改革增加公共服务供给。鼓励更多社会资本参与教育、医疗、文化、养老等供给。深化医疗卫生体制改革，全面推广罗湖医改模式，完善分级诊疗体系，建立家庭医生服务标准和签约服务激励机制，实施公立医院人事制度综合配套改革，开展医养结合试点，创建国家中医药综合改革试验区，推动从“治疾病”向“促健康”转变。深化教育综合改革，创新高等教育办学体制机制，推进中小学校长职级制，探索建立产教融合的职业教育体系。完成机关事业单位养老保险制度改革。

　　（二）实施新一轮创新发展战略布局，加快建设国际科技、产业创新中心。抢抓新一轮科技和产业变革战略机遇，制定实施国际科技、产业创新中心建设方案，着力推动创新生态再优化、创新能力再突破、创新经济再升级，强化全国经济中心城市和国家创新型城市地位。今年起实施“十大行动计划”。一是布局十大重大科技基础设施。立足提升源头创新能力，力争在信息科学与安全、生命科学与健康等领域建设国家实验室，加快推进未来网络实验设施建设和深圳超算中心扩容，规划建设国家基因库二期、生物医学大数据等重大科技基础设施。二是设立十大基础研究机构。重点在数学、医学、脑科学、新材料、数字生命、数字货币、量子科学、海洋科学、环境科学、清洁能源等领域谋划建设10个基础研究机构，开展前沿科学探索、关键技术研发、高端人才培养等，强化创新的基础支撑。今年启动4个研究机构建设。三是组建十大诺贝尔奖科学家实验室。发挥诺贝尔奖科学家的引领带动作用，在化学、医学、光电等领域，规划建设10个由诺贝尔奖获得者领衔的实验室，力争今年新建2个以上。新增国家省市级重点实验室、工程实验室、工程研究中心、企业技术中心等创新载体100家以上。四是实施十大重大科技产业专项。集中资源加大研发、产业化、应用等全链条支持力度，加快在石墨烯、微纳米、机器人、5G移动通信、金融科技、VR/AR等10个领域出台专项支持计划，抢占前沿技术产业化先机，增强创新型经济发展后劲。五是打造十大海外创新中心。着眼全球加大开放创新布局力度，出台专项计划，支持企业和机构在美国、欧洲、加拿大等创新资源高度密集地区，规划建设10个海外创新中心，构建国际协同创新平台，集聚全球创新能量。今年启动4个海外创新中心建设。六是建设十大制造业创新中心。落实“互联网＋”和“中国制造2025”行动计划，在机器人、3D打印、新型显示、精准医疗、新能源汽车、海洋工程装备等领域规划建设10个创新中心，提升高端制造、精密制造、智能制造能力水平。今年启动3个创新中心建设。七是规划建设十大未来产业集聚区。坚持科学规划、前瞻布局、联动发展，在生命健康、海洋经济、航空航天等未来产业领域，加快建设10个集聚区，培育若干千亿级产业集群。八是搭建十大生产性服务业公共服务平台。重点在创新设计、品牌培育、知识产权运营、检验检测认证等领域，打造10个重大平台，提升生产性服务业发展能级，助推制造业转型升级。九是打造十大“双创”示范基地。加快国际创客中心建设，高水平建设深圳湾创业广场等十大“双创”示范基地，强化创新、创业、创投、创客“四创联动”，当好“双创领跑者”。新增众创空间40家以上、创客服务平台10家以上。十是推进十大人才工程。实施“院士引进工程”“创新领军人才集聚工程”“优秀大学毕业生引进计划”等，加大力度推进“孔雀计划”“双百苗圃计划”“高技能人才振兴计划”，全年引进院士10名左右，孔雀团队30个以上，海外高层次人才400名以上。

　　（三）加快培育新增长点，促进经济更高质量稳定增长。始终坚持发展第一要务，统筹适度扩大总需求和积极增加有效供给，继续实施增量优质、存量优化“双优工程”，协同推进产业、区域“双转型”，以中高端发展支撑中高速增长。一是释放需求新潜力。扩大工业、基础设施、民生工程等高质量投资，加快华星光电第11代液晶面板、中芯国际12英寸芯片、日东电工偏光片、比亚迪云轨试验线、国际会展中心等重大项目建设，全年固定资产投资增长20%以上。发展文体、旅游、健康、信息、邮轮游艇等新兴消费，加快中国国际消费电子展示交易中心建设，推进华强北、东门转型升级和小梅沙片区改造，完善口岸消费环境，创建罗湖国家消费型经济综合改革试验区，全年社会消费品零售总额增长8%左右。加大引进外资力度。推进外贸稳份额、优结构、提质量，加快出口加工区、保税区等转型升级，开展服务贸易创新发展试点，一般贸易、服务贸易比重分别提高至45%和21%。二是培育优质产业新增量。创新战略性新兴产业和未来产业支持政策，全年支持产业项目1000个以上，产业规模超过3.1万亿元。新增国家级高新技术企业2000家。积极创建国家军民融合创新示范区。大力发展创新金融、现代物流和专业服务等现代服务业，加快布局金融科技，加强金融产品和服务创新，新增持牌法人金融机构15家以上，金融业增加值增长14%；提升科技服务业发展水平，推进国家技术转移南方中心建设，全年交易额550亿元以上。进一步增强高交会、文博会、IT和BT领袖峰会、电子信息博览会等展会实效。推动黄金珠宝等优势传统产业向价值链高端延伸，继续办好国际时装周、时尚家居设计周、工业设计大展等活动。积极发展“高精尖”现代农业。三是发展壮大新业态。抢占信息经济发展制高点，突出跨界融合、交叉渗透，以互联网思维、云计算架构、大数据支撑，推动供应链重组、产业链重构和价值链重塑，发展数字经济、分享经济、平台经济，创建信息经济示范区和大数据综合试验区。支持跨境电商、外贸综合服务企业等新业态发展，跨境电商进出口增长15%。四是打造区域新增长极。开展特区一体化五年攻坚行动，加大对原特区外地区的支持力度，全年政府投资80%以上投向原特区外及特区一体化项目。实施东进战略，规划建设东部过境、春风隧道、大鹏第二通道等一批重大项目，推进大梧桐新兴产业带、坪山高技术产业园区等建设，高水平打造东部创新产业带和东部黄金海岸旅游带。统筹实施中轴提升和西部优化战略，加快深圳湾超级总部基地、北站商务中心区、空港新城等重点区域开发，全年完成投资1200亿元以上，增长30%。

　　（四）主动服务“一带一路”倡议，以共建粤港澳大湾区构建开放发展新格局。始终在全球坐标系下谋划开放布局，把携手建设粤港澳大湾区作为重塑开放发展新优势的重大战略，以创新投资贸易规则为突破口，以构建新的战略通道为抓手，以区域协同联动为支撑，实现更高水平内外联动和双向开放。一是拓展深港澳紧密合作新空间。以香港回归20周年为契机，全方位加强与香港在经贸、金融、教育、科技等各领域合作，谋划启动一批重大合作项目，加快落马洲河套地区深港合作开发，建设深港创新及科技园，推进莲塘/香园围口岸、深圳河四期治理等重点项目建设，推动皇岗、沙头角口岸重建和深圳湾口岸周边交通改造，探索深港VC/PE通。加强与澳门在文化创意、特色金融、旅游等领域合作，联手开拓葡语国家市场。二是打造前海蛇口自贸片区高水平对外开放门户枢纽。突出制度创新核心任务，对照最高标准，加大投资贸易规则、跨境金融等创新力度，形成更多可复制可推广的创新成果。坚持依托香港、服务内地、面向世界，加快前海深港现代服务业合作区建设，打造深港基金小镇，全年新引进港企2000家以上、香港青年创新创业团队80余个。推进桂湾金融先导区等建设提速，全年完成固定资产投资420亿元以上。构建“大前海”发展格局，促进前海与周边区域协同联动，大力拓展对外合作新通道，推动自贸片区加快成为对外开放门户枢纽。三是建设“一带一路”重要枢纽。深化与沿线国家经贸、投资等合作，拓展驻外经贸代表机构服务职能，推进中白物流园、印尼深圳产业园等境外园区建设，支持企业参与巴林、斯里兰卡、吉布提、土耳其等国家港口建设运营。加强与沿线节点城市的合作，积极参与新疆丝绸之路经济带创新驱动发展试验区和中巴经济走廊建设，携手开拓中亚、南亚市场。办好2017年国际产能合作论坛暨中国对外投资合作洽谈会。四是构建开放发展的战略通道。立足在更大范围配置资源，建设国际航空枢纽、全国综合铁路枢纽和世界级集装箱枢纽港，打造“海陆空铁”全方位立体化战略通道。规划建设第三跑道、卫星厅、T4航站楼，新开通5条国际航线。推进盐田港东港区20万吨级泊位和西部出海航道建设，强化海铁联运。加快赣深客专、深茂铁路深圳至江门段、穗莞深城际线和深中通道、外环高速等建设，促进交通基础设施互联互通，推动深莞惠“3＋2”经济圈等深度融合发展。

　　（五）着力打造绿色发展示范市，建设更加宜居的绿色家园。碧水蓝天是广大市民的共同期待，良好生态环境是最普惠的民生。我们要始终坚持环境就是生产力、竞争力，持续加大环保投入，狠抓突出环境问题整改，不断巩固提升环境质量，努力为市民提供高品质的生态环境供给。一是扎实开展治水提质行动。坚持流域治理和流域开发相结合，进一步健全“河长制”，加强茅洲河、坪山河、龙岗河、观澜河、深圳湾等治理，完成茅洲河界河段综合整治，基本消除建成区黑臭水体。实施污水处理厂提质工程，建成沙井水质净化厂二期，提标改造8座水质净化厂，开工松岗二期、洪湖、布吉水质净化厂，新增污水管网1500公里，创建排水达标小区1000个，整治河道119公里，治理59个内涝点。推进国家海绵城市试点。建设东部湾区国家生态文明先行示范区。二是高标准打造一流环保基础设施。瞄准世界最先进水平，采用最领先技术，加快建设东部环保电厂、老虎坑三期、妈湾能源生态园，推进现有环保设施提标升级和餐厨垃圾处理设施建设，完成114座垃圾转运站改造，推动污泥减容减量。三是持续提升大气等环境质量。加快实施清洁柴油车计划，实现公交大巴全部纯电动化，新推广新能源汽车2万辆，新建充电桩2.5万个。推进绿色港口建设，新建成覆盖9个泊位的岸电设施。推广装配式建筑和合同能源管理，加强建筑废弃物源头减排和综合利用，新增绿色建筑面积800万平方米。加大挥发性有机物和扬尘污染治理力度。新建、改建公园55个，推进航天公园、人才公园等特色主题公园建设，建成100公里“远足径”和深圳湾滨海休闲带西段。创建国家森林城市，办好第19届国际植物学大会。实施土壤环境保护和质量提升工作方案。开展城市噪声污染整治。全面推行垃圾分类和减量，加快完善全链条、全覆盖的分类制度和处理系统，达标小区占比提高至40%以上。

　　（六）突出精细化智能化，提高城市建设管理质量。巩固提升城市管理治理成果，出台加强城市规划建设管理工作的意见，科学编制第四轮城市总体规划，推动城市现代化国际化水平再上新台阶。一是加快提升建设管理标准。瞄准国际一流，制定完善城市设计、建筑设计等标准规范，系统梳理提升基础设施、建筑工程、市容市貌等各方面标准，打造精品建筑、精致街区、精美公园，着力提升城市品味品质。推进城市管理精细化，加强市政设施等管养维护，完善机场、车站、口岸等区域标识标牌和无障碍设施。二是建设适度超前的基础设施。加快轨道交通三期6、8、10号线等建设，开工四期12、13、14、16号线，建成龙华有轨电车示范线。启动盐港东立交、梅观高速市政化等改造工程，推进坪盐通道、南坪三期、坂银通道等建设提速。开展西江引水前期工作，建成公明、清林径两大水资源调蓄工程，完成108个社区供水管网、150个小区优质饮用水入户改造。建设30公里地下综合管廊，建成抽水蓄能电站、滇西北至广东特高压直流输电深圳段工程。三是优化拓展城市发展空间。坚持陆海统筹、科学用海、依法治海，注重开发保护并举，建设海洋综合管理示范区和大鹏新区国家级海洋生态文明示范区，实施“蓝色海湾”整治行动，不断强化滨海城市特色。加强城市立体开发，推进地下空间开发利用和互联互通。保持违建整治高压态势，完善清拆控停措施，确保违建“零增量”，全年拆除消化违建比上年增加一倍以上。优化基本生态控制线和法定图则管理，深入推进拓展空间十大专项行动，加快土地整备利益统筹试点，全年城市更新供应土地2.5平方公里、整备释放用地12平方公里，完成旧工业区综合整治100万平方米。四是打造新型智慧城市标杆市。完善信息基础设施，建设国际一流的信息港，新建移动通信基站1.5万座，实现光纤入户率超过90%，推进IPv6规模化商用。强化信息资源深度整合，打破信息孤岛和数据壁垒，建成统一的城市综合信息数据平台，推进各类数据实时接入、开放共享，实施智能交通、智慧教育、智慧医疗等信息惠民工程。加强网络信息安全建设，保障数据安全和个人隐私。

　　（七）牢固树立底线思维和风险意识，大力提升安全发展水平。安全是发展之基。要把防风险摆在突出位置，加强各类风险预警研判和隐患排查治理，提高防范化解和应对处置能力，牢牢守住安全发展的底线。一是加强金融、房地产风险防控。落实国家房地产市场调控政策，坚持“房子是用来住的、不是用来炒的”的定位，完善房地产市场调控长效机制，合理增加住宅用地供应，有效引导市场预期，促进房地产市场平稳健康发展。加大对金融风险的监测防范力度，完成互联网金融风险专项整治，严厉打击非法集资等违法违规行为，确保不发生区域性系统性金融风险。二是强化安全生产监管。坚持标本兼治、源头防范、系统治理，严格落实安全生产责任，坚决防范重特大事故。建立健全风险管控和隐患排查治理双重预防机制，全面完成风险点、危险源排查评估，进一步完善公共安全白皮书。实行企业安全生产不良记录“黑名单”制度。深化危险化学品、油气管道、建筑施工、道路运输等专项整治，改造250公里老旧燃气管道，推动物业小区老化电线更换。推进部九窝、水径余泥渣土受纳场等重点安全隐患整治，加快二线“插花地”拆除重建，加大危险房屋排查处置。提高应急装备水平，推动应急管理由救灾响应型向防灾准备型转变。加强消防安全等宣传，增强全社会安全意识。三是推进“平安深圳”建设。加快建立立体化、信息化社会治安防控体系，落实人防、物防、技防措施，严厉打击网络电信诈骗、“两抢一盗”和恶性刑事犯罪等，加强社会矛盾排查化解，确保社会和谐稳定。

　　（八）坚持以人民为中心的发展思想，持续增进民生福祉。要时刻把人民的衣食住行和冷暖安危记在心上、扛在肩上、抓在手上，从市民需求出发，从解决突出问题入手，多为市民办实事、做好事、解难事，努力让市民有更多实实在在的获得感。全年安排九大类民生支出2700亿元以上，完成12项重大民生工程投资300亿元，办好119件年度民生实事和一大批民生“微实事”。一是扩大基础教育学位供给。新改扩建义务教育学校30所，建成人大附中深圳学校、第八高级中学等学校，新增普惠性幼儿园100所，增加幼儿园学位2万个、公办中小学学位4.7万个。推进有条件的中小学提供在校午托服务。二是提升基层医疗服务能力。组建基层医疗集团，构建区域医疗卫生服务共同体。推动社区、商务区等增设便民医务室，在300家社康中心设立专科医生工作室，增加慢性病常用药物等配置。全面推广家庭医生签约服务，新增签约人数30万名。三是增加优质医疗服务供给。着力打造国际化医疗中心，深入实施“三名工程”，引进高水平医学团队50个，新建名医诊疗中心3家以上。推进59个医院新改扩建工程，建成中山大学附属第七医院一期、深圳大学附属医院、孙逸仙心血管医院新院区，开工建设市第二儿童医院、新华医院，新增病床2000张、执业医师2000名。推动精准医院、健康科学院和质子治疗中心建设，新增无创产前和宫颈癌基因检测两项公共卫生服务项目。四是加大住房保障力度。新开工及筹集人才和保障性住房8万套、供应4.5万套，同步推进教育、医疗、交通、养老等配套设施建设，完善分配、定价、封闭流转、退出等管理制度。五是着力治理交通拥堵。实施一批交通专项整治计划，加快高新区、梅林关、布吉关、盐田港后方陆域等重点区域交通拥堵整治，推进综合立体停车场和立体机械停车库建设，打通51条断头路。加强地铁、公交、慢行交通的接驳，增开30条社区微巴线路，促进共享单车健康发展，着力解决出行“最后一公里”问题。六是加强食品药品安全监管。坚持严字当头，深入实施食品安全示范工程，加快提高安全标准、检测能力、抽检频次，健全食品安全全程追溯体系，严厉打击食品药品违法行为，切实强化源头严防、过程严管、风险严控，实现食品和食用农产品抽检覆盖率提高至每千人9批次，努力守住“舌尖上的安全”。完善药品监管机制，保障市民用药安全。七是改善老旧小区和城中村环境。采取市区联动、政社企共担等方式，推进电梯加装和更新维护，整治脏乱差问题，优化路面、绿化等环境，增设若干便民文体设施。八是优化公共场所免费WLAN服务。统筹推进各区和地铁、公交等公共场所免费WLAN统一认证接入，加快实现一次登录认证、全市通用，改善市民接入体验。九是推进文体惠民。全面实施深圳“文化创新发展2020”，大力发展文化创意产业，着力提升文化软实力。加快推进新科技馆、美术馆新馆等项目，探索建立当代艺术品交易中心，举办“一带一路”国际音乐季、科技影视节、深圳读书月等文体活动，推动公共体育场馆免费或优惠向市民开放。培育和践行社会主义核心价值观，开展市民文明素养提升行动，争创第五届全国文明城市。十是推动高等教育开放发展。支持深圳大学、南方科技大学加快建设高水平大学，开工建设中山大学深圳校区，完成香港中文大学（深圳）一期工程，争取清华伯克利深圳学院、深圳墨尔本生命健康工程学院等特色学院获批筹设，推进北京大学、清华大学等一批名校深圳校区加快落地。借鉴德国、瑞士等国家先进经验，探索“双元制”职业教育新模式，加大职业培训力度，推进深圳技术大学、中德先进制造技师学院等规划建设。十一是积极发展养老等社会事业。稳步提高社会保障水平。构建和谐劳动关系，新增就业8万人。建立特困人员救助供养机制。扩大优质养老服务供给，新增养老床位1000张以上。开展扶贫济困日、公益慈善月等活动，引导民间慈善和互联网募捐健康发展。加强社会治理和基层基础建设，推动物业管理条例修订，完成社区居委会换届选举，推进社区党群服务中心规范化建设，办好2017国际社会工作亚太区域联合大会。

　　（九）积极践行“两个大局”战略思想，坚决做好对口支援、扶贫协作和对口合作。始终秉持“感恩改革开放、回报全国人民”的情怀，认真落实中央和省精准扶贫、精准脱贫部署，携手对口地区共同打赢脱贫攻坚战，更好地服务全国全省区域协调发展。全年安排各类帮扶资金约70亿元。一是继续高标准推进对口支援工作。加强援疆援藏，着力抓好喀什“一城一园一校一区”、西藏非物质文化遗产博物馆等项目建设，帮助新疆塔县、西藏察隅县贫困人口加快脱贫。积极推进对口支援重庆巫山、四川甘孜工作。二是加大全面对口帮扶河源、汕尾力度。主动服务粤东西北振兴发展战略，突出民生帮扶、产业共建，助推323个相对贫困村精准脱贫，帮助两市各新建一家三级医院，加快深汕特别合作区、深河产业园及10个区县共建产业园建设。三是扎实开展对口协作等工作。推进与广西百色、河池扶贫协作，在移民搬迁、产业合作、基础设施建设、教育文化卫生等方面谋划实施一批帮扶项目。继续做好在深就业的湖南湖北三县贫困人口跟踪服务工作。此外，启动并做好与哈尔滨对口合作。

　　（十）坚决落实全面从严治党要求，扎实推进从严治政。坚持党的领导是做好政府工作的根本保证。要牢固树立“四个意识”，更加自觉在思想上政治上行动上同以习近平同志为核心的党中央保持高度一致，始终坚持在党的领导下开展工作，把全面从严治党要求落实到政府工作的各个方面，切实做到从严治政、依法行政、廉洁从政，不断提高政府治理能力现代化水平。一是加强政府系统党的建设。严格落实全面从严治党主体责任，严格执行党内政治生活若干准则、党内监督条例、党组工作条例等党内法规，以更高标准加强党风廉政建设。深入落实中央八项规定精神，进一步巩固整治“四风”成果。坚持无禁区、全覆盖、零容忍，始终保持反腐败高压态势，严肃查办各类违法违纪行为，进一步规范行政权力运行。二是建设一流法治政府。落实推进法治建设第一责任人制度，自觉接受市人大及其常委会的法律监督、工作监督和市政协的民主监督，认真执行市人大及其常委会决议决定，坚持重大事项向市人大及其常委会报告、向市政协通报制度，健全与人大代表、政协委员的沟通联络机制，高质量办好建议提案。推进政府决策、执行、管理、服务、结果“五公开”，加强行政监察、审计监督，更加主动地接受全社会监督，及时整改问题，不断健全制度、堵塞漏洞。清理修改政府规章和规范性文件。改革完善城管、环保执法体制机制，强化安全生产、违法建筑、环境保护、食品药品安全等重点领域的行政执法。三是提升政府服务管理效能。推进“互联网＋政务服务”，扎实开展“一门式、一网式”服务模式改革，建设统一的政务服务大数据库、电子证照库，优化重构政府运作流程，进一步精简环节、减少程序，清理隐性审批和变相审批，强化网上审批、并联审批和集中联审，实现效率再提升、服务再优化。四是强化工作落实。弘扬“马上就办、真抓实干”的作风，以“钉钉子”精神抓落实、抓攻坚，强化督查督办、考核问责和正向激励，健全有利于“想干事、敢干事”的体制机制，确保中央、省和市各项决策部署不折不扣落实到位。

　　各位代表，特区肩负着特殊使命，承载着特别期望。让我们更加紧密地团结在以习近平同志为核心的党中央周围，紧紧依靠全市人民，解放思想、真抓实干，不忘初心、继续前进，加快建设现代化国际化创新型城市和国际科技、产业创新中心，勇当“四个全面”排头兵、“四个自信”践行者，以深圳经济特区特别的作为，更好地彰显中国特色社会主义制度的优越性，以优异的成绩迎接党的十九大和省第十二次党代会胜利召开，为实现“三个定位、两个率先”目标和中华民族伟大复兴的中国梦作出更大贡献！
